# Concurrent presentation of acute lymphoblastic leukemia and bullous pemphigoid: a rare case report

**DOI:** 10.1093/omcr/omae173

**Published:** 2025-01-18

**Authors:** Yusuf Haz Condeng, Rahmawati Minhajat, Andi Makbul Aman, Haerani Rasyid, Syakib Bakri, Harun Iskandar

**Affiliations:** Department of Internal Medicine, Faculty of Medicine, Hasanuddin University, Jalan Perintis Kemerdekaan KM. 11, Makassar, South Sulawesi 90245, Indonesia; Department of Internal Medicine, Faculty of Medicine, Hasanuddin University, Jalan Perintis Kemerdekaan KM. 11, Makassar, South Sulawesi 90245, Indonesia; Department of Internal Medicine, Faculty of Medicine, Hasanuddin University, Jalan Perintis Kemerdekaan KM. 11, Makassar, South Sulawesi 90245, Indonesia; Department of Internal Medicine, Faculty of Medicine, Hasanuddin University, Jalan Perintis Kemerdekaan KM. 11, Makassar, South Sulawesi 90245, Indonesia; Department of Internal Medicine, Faculty of Medicine, Hasanuddin University, Jalan Perintis Kemerdekaan KM. 11, Makassar, South Sulawesi 90245, Indonesia; Department of Internal Medicine, Faculty of Medicine, Hasanuddin University, Jalan Perintis Kemerdekaan KM. 11, Makassar, South Sulawesi 90245, Indonesia

**Keywords:** hematologic malignancies, lymphoproliferative disorders, hematologic-dermatologic intersection

## Abstract

Historically, adolescents and young adults diagnosed with acute lymphoblastic leukemia (ALL) have faced lower survival rates compared to children with the same illness. Bullous pemphigoid (BP), a rare autoimmune skin disorder, poses unique challenges when occurring alongside hematologic malignancies. A 23-year-old male with ALL-L1 diagnosis who developed bullous pemphigoid in this report. The patient exhibited typical ALL-L1 symptoms, including constitutional manifestations and signs of bone marrow compromise. Dermatological assessment revealed extensive edematous urticaria-like plaques, erosions, excoriations, crusts, and a hemorrhagic bulla. Severe thrombocytopenia was evident in laboratory tests, with histopathological examination confirming bullous pemphigoid. Despite aggressive treatment, including platelet transfusions, the patient’s condition worsened. This case emphasizes the critical need for timely diagnosis and intervention in patients with concurrent hematologic and dermatologic conditions, as mortality rates may surpass those in BP patients without comorbidities.

## Introduction

Survival rates for adolescents and young adults diagnosed with acute lymphoblastic leukemia (ALL) have historically been lower compared to those observed in children diagnosed with the same disease [1]. In 2016, the American Cancer Society documented approximately 6590 newly detected cases of ALL, resulting in over 1400 deaths. According to more recent data, the incidence of ALL remains significant, with survival disparities continuing to be a concern. ALL has a bimodal distribution, with peaks in childhood and around the age of 50 [2]. Based on the research by Schulze et al. and Kridin et al., there is a notable association between pemphigus and hematologic malignancies. Schulze et al. found that 3.9% of pemphigus patients had hematologic malignancies [3]. Herein, we report a case of acute lymphoblastic leukemia (ALL-L1) presenting with bullous pemphigoid.

## Case report

**Figure 1:**
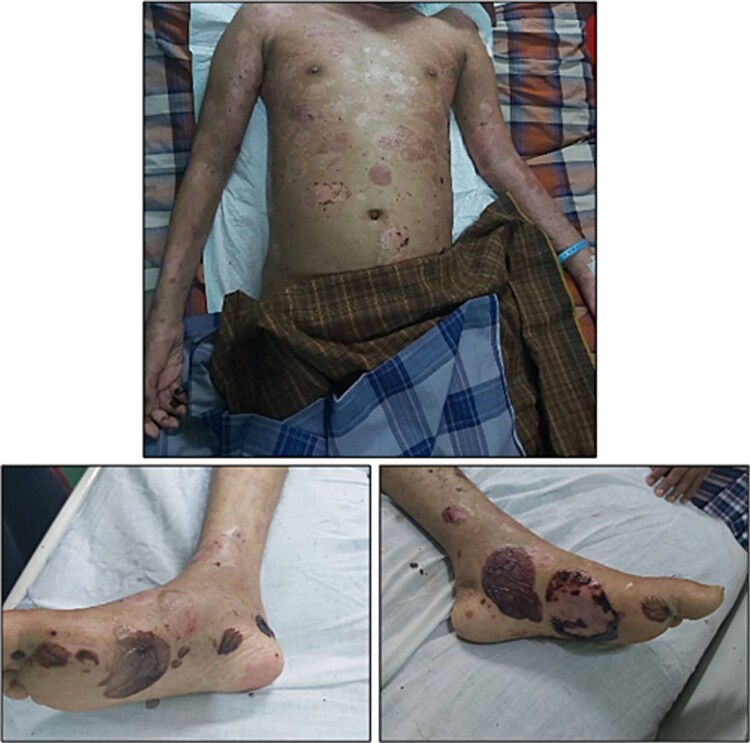
The initial presentation upon admission revealed extensive edematous urticaria-like plaques, accompanied by desquamation, erosion, excoriation, and crusting, along with a ruptured hemorrhagic bulla located on the anterior body and left leg.

Mr. RR, a 23-year-old male, presented at the emergency room reporting a week-long period of weakness that had worsened prior to admission, significantly impeding his daily activities. He described a four-month history of recurring, small fluid-filled sacs on the skin. These lesions evolved from reddish patches to rupturing and oozing, accompanied by itching but no pain or fever. He denied experiencing chest pain, shortness of breath, or vomiting but mentioned recent episodes of nausea. Upon examination, he appeared moderately unwell, with normal vital signs and pale conjunctiva. Physical assessment revealed palpable splenomegaly (Schuffner 6) but no lymph node enlargement or cardiac murmurs. Dermatologic examination revealed oedematous urticaria-like plaques, scaling, erosions, excoriations, and crusts all over the body, as well as a ruptured hemorrhagic bulla on the anterior body and left leg (Fig. 1).

**Figure 2:**
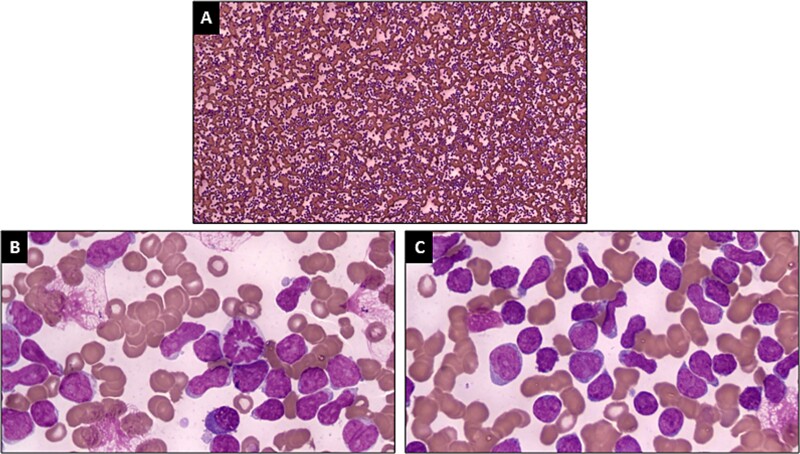
Bone marrow aspiration at diagnosis (**A–D**). (A) 100× magnification. (**B**) and (**C**) 1000× magnification with emersion oil.

**Figure 3:**
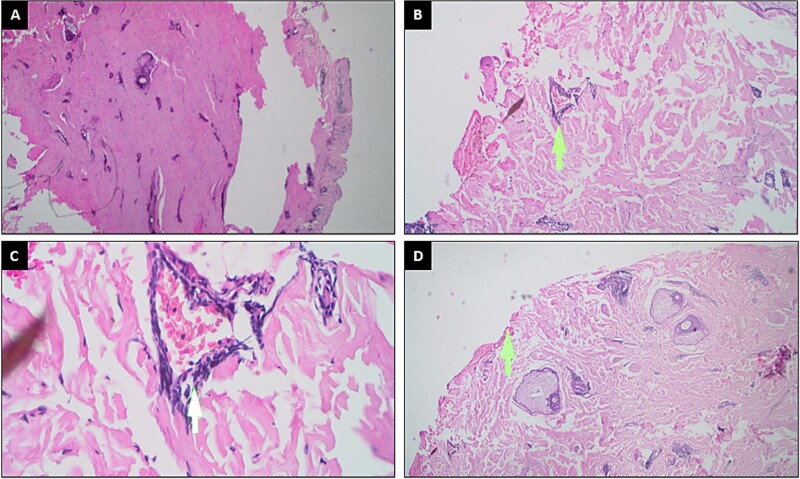
Histopathological analysis of the ruptured hemorrhagic bulla on the anterior trunk on the eighth day of treatment, using hematoxylin and eosin stain, yielded the following observations (**A–D**). (**A**) Visualized at 4× magnification. (**B**) Enlarged to 10×, highlighting perivascular lymphocytes indicated by a yellow arrow. (**C**) Enhanced to 40×, with perivascular lymphocytes marked by a white arrow. (**D**) Presented at 4× magnification, focusing on a non-optimal area due to the absence of the epidermis and bulla base impression, as indicated by a yellow arrow.

Complete blood test showed that the White Blood Cell Count (WBC) was 23 × 10^9^/l, the Absolute Neutrophil Count (ANC) was 644 cells/μl, the Hemoglobin (Hb) was 99 g/, and the Platelet Count (Plt) was 28 × 10^9^/l. Bone marrow aspiration consistent with acute lymphoblastic leukemia-L1, led to the diagnosis of acute lymphoblastic leukemia in the patient (Fig. 2). The decision for chemotherapy has not been approved by the patient, so patient only received corticosteroids and supportive therapy. At the onset of therapeutic intervention, the patient presented with erythematous patches accompanied by vesicular lesions spanning almost the entirety of his body. Subsequently, platelet concentrate transfusions were administered on the fourth and sixth days of treatment, with a total of eight bags administered on each occasion. On the eighth day of treatment, a histopathological exam showed that the patient had bullous pemphigoid because there was necrotic epithelium and chronic inflammatory cells (Fig. 3). The decision to forgo direct immunofluorescent assay (DIF) was predicated upon the patient’s existing thrombocytopenia and heightened vulnerability to bleeding complications. Subsequently, on the twelfth day of treatment, the patient underwent a transfusion of one unit of packed red cells (PRC). Following this, on the thirteenth day of treatment, the patient received two units of PRC and eight units of platelet concentrates. On the fourteenth day of treatment, the patient experienced an abrupt loss of consciousness, preceded by a severe headache and anisocoric pupils. Laboratory findings revealed a post-transfusion platelet count of 7000/ul after eight bags of platelets were administered. Despite continued therapeutic interventions.

## Discussion

The French-American-British (FAB) classification identified the patient as having ALL subtype L1. ALL subtype L1 is characterized by blast cells that are more uniform in appearance, have a regular nucleus, and contain minimal cytoplasm [4]. Bullous pemphigoid initially presents with widespread eczematous, pruritic, and urticaria-like lesions. Over time, these lesions progress to form tense bullae or blistering lesions, which are usually filled with clear fluid. These lesions are predominantly found on the trunk, extremity flexor surfaces, and axillary regions [5]. Subepidermal blisters, accompanied by a perivascular inflammatory infiltrate containing eosinophils, are among the characteristic findings. Eosinophilic spongiosis is a distinctive feature and is highly indicative of bullous pemphigoid [6].

A comprehensive record-linked study conducted in England from 1999 to 2011 identified an increased incidence of bullous pemphigoid in patients diagnosed with lymphoid leukemia compared to a control cohort [7]. In cross-sectional analyses, a notable association emerged between bullous pemphigoid (BP) and hematologic malignancies. Despite initial findings from a single cohort study indicating potential links to specific cancer subtypes, thorough pooled analyses failed to validate these observations. Researchers uncovered intriguing indications of a plausible relationship with hematologic malignancies, but a definitive overarching correlation between BP and malignancy remained elusive [8].

Lymphoproliferative disorders cause significant alterations in the immune system due to autoantibodies generated by neoplastic cells. The primary treatment for dermatoses associated with malignancies typically involves systemic steroids and immunosuppressants. However, if the underlying malignancy remains untreated, therapies targeting bullous pemphigoid alone are insufficient to improve the prognosis. In rare instances, dermatoses may indicate a potentially unfavorable prognosis for the associated hematological malignancy [9]. The primary differential diagnosis is paraneoplastic pemphigus, a rare condition that is often connected with lymphoproliferative neoplasms (84% of the time). These can be chronic lymphocytic leukemia (CLL), non-Hodgkin’s lymphoma, Castleman’s disease, or thymoma [10].

A unique aspect of this case is the patient’s young age. Bullous pemphigoid is typically reported in patients with concomitant hematological disorders, particularly in older populations. The limitations of this case report merit scrutiny, especially the lack of a direct immunofluorescent assay (DIF) due to the patient’s thrombocytopenia. The absence of DIF, a cornerstone in diagnosing bullous pemphigoid, may have impacted diagnostic certainty, relying solely on histopathological examination. Clinicians should prioritize the use of DIF where possible, even in challenging clinical scenarios, to ensure diagnostic accuracy. The management plan should also have included an early referral to a hematologist-oncologist in collaboration with a dermatologist. Given the patient’s initial refusal to undergo chemotherapy, it would have been prudent to prepare for chemotherapy once the patient’s condition stabilized. The initiation of chemotherapy is critical not only for controlling the leukemia, but also for potentially mitigating the progression of BP, as untreated malignancies can exacerbate autoimmune conditions.

## Summary

This case of a young adult with acute lymphoblastic leukemia (ALL) and bullous pemphigoid (BP) shows that delaying chemotherapy in favor of corticosteroids alone may make treatment less effective and speed up the patient’s decline. A multidisciplinary approach, involving both dermatologists and hematologist-oncologists, is essential for optimizing patient outcomes. Early chemotherapy, once the patient’s condition allows, is crucial for managing both the leukemia and the associated autoimmune disorder. This case underscores the need for timely, coordinated care in patients with complex dual diagnoses.

## Consent

Informed patient consent was obtained.

## Guarantor

Yusuf Haz Condeng (YHC).
